# Supplementary material for: Complexome profiling of the Chlamydomonas psb28 mutant reveals TEF5 as an early PSII assembly factor
Source: Plant Cell. 2025 Mar 17;37(6):koaf055. doi: 10.1093/plcell/koaf055 (PMC12164590; doi:10.1093/plcell/koaf055)
Supplement: koaf055_Supplementary_Data [file koaf055_supplementary_data.zip › psb28_interactive.zip!/plots/intensity/qualityPlots/peptides/PGR5_Cre05.g242400.t1.2.html]

### Cre05.g242400.t1.2

Trivial name: PGR5  
SeqCoverage [median]: 35.50  
Mass: 14.86 kDa  
  
 

| Sequence | MissedCleavage | Mass [Da] | wtIntensity | muIntensity | Score |
| --- | --- | --- | --- | --- | --- |
| AISYHSQIIK | 0 | 1158.64 | 0.00 | 924.31 | 44.20 |
| ATTGPFAPLVIVVR | 0 | 1439.85 | 21862.37 | 17663.45 | 225.01 |
| GAMGEKPFNNFR | 1 | 1366.65 | 13933.80 | 28228.78 | 126.19 |
| LLGVDNK | 0 | 757.43 | 0.00 | 4669.04 | 124.34 |
| LLGVDNKQVQGVIR | 1 | 1537.89 | 386.79 | 0.00 | 52.55 |
| QVQGVIR | 0 | 798.47 | 14313.53 | 19822.19 | 150.07 |
|  |  |  | 50496.5 | 71307.8 |  |
